# Distributional regression in clinical trials: treatment effects on parameters other than the mean

**DOI:** 10.1186/s12874-022-01534-8

**Published:** 2022-02-27

**Authors:** Gillian Z. Heller, Kristy P. Robledo, Ian C. Marschner

**Affiliations:** NHMRC Clinical Trials Centre, University of Sydney, Locked bag 77, Camperdown, NSW 1450 Australia

**Keywords:** Distributional regression, Clinical trials, Treatment effect, Gamlss

## Abstract

**Background:**

The classical linear model is widely used in the analysis of clinical trials with continuous outcomes. However, required model assumptions are frequently not met, resulting in estimates of treatment effect that can be inefficient and biased. In addition, traditional models assess treatment effect only on the mean response, and not on other aspects of the response, such as the variance. Distributional regression modelling overcomes these limitations. The purpose of this paper is to demonstrate its usefulness for the analysis of clinical trials, and superior performance to that of traditional models.

**Methods:**

Distributional regression models are demonstrated, and contrasted with normal linear models, on data from the LIPID randomized controlled trial, which compared the effects of pravastatin with placebo in patients with coronary heart disease. Systolic blood pressure (SBP) and the biomarker midregional pro-adrenomedullin (MR-proADM) were analysed. Treatment effect was estimated in models that used response distributions more appropriate than the normal (Box-Cox-t and Johnson’s *S*_*u*_ for MR-proADM and SBP, respectively), applied censoring below the detection limit of MR-proADM, estimated treatment effect on distributional parameters other than the mean, and included random effects for longitudinal observations. A simulation study was conducted to compare the performance of distributional regression models with normal linear regression, under conditions mimicking the LIPID study. The **R** package **gamlss** (Generalized Additive Models for Location, Scale and Shape), which implements maximum likelihood estimation for distributional regression modelling, was used throughout.

**Results:**

In all cases the distributional regression models fit the data well, in contrast to poor fits obtained for traditional models; for MR-proADM a small but significant treatment effect on the mean was detected by the distributional regression model and not the normal model; and for SBP a beneficial treatment effect on the variance was demonstrated. In the simulation study distributional models strongly outperformed normal models when the response variable was non-normal and heterogeneous; and there was no disadvantage introduced by the use of distributional regression modelling when the response satisfied the normal linear model assumptions.

**Conclusions:**

Distributional regression models are a rich framework, largely untapped in the clinical trials world. We have demonstrated a sample of the capabilities of these models for the analysis of trials. If interest lies in accurate estimation of treatment effect on the mean, or other distributional features such as variance, the use of distributional regression modelling will yield superior estimates to traditional normal models, and is strongly recommended.

**Trial registration:**

The LIPID trial was retrospectively registered on ANZCTR on 27/04/2016, registration number ACTRN12616000535471.

**Supplementary Information:**

The online version contains supplementary material available at 10.1186/s12874-022-01534-8.

## Background

The classical linear model is widely used in the analysis of clinical trials with continuous outcomes. However when the required model assumptions are not met, estimates of treatment effect can be inefficient and biased. Since Nelder and Wedderburn’s [1] seminal paper introducing generalized linear models (GLMs), there has been a revolution in the development of regression methodology, enabled by an accompanying exponential increase in computing power over the same period. The current state-of-the-art “distributional regression” framework, first proposed by [2] as Generalized Additive Models for Location, Scale and Shape (GAMLSS), accommodates any computable parametric response distribution; the facility for modelling any distributional parameter (not just the mean), smooth terms for continuous covariates; and random effects for modelling clustered (e.g. longitudinal) observations. Within this rich environment, the analyst has the ability to model trials outcomes using appropriate assumptions, resulting in estimates of treatment effect which should be efficient and asymptotically unbiased.

The use of distributional regression in clinical trials has broader motivations than efficient and unbiased statistical modelling. The mean of a biomarker may not be the only feature of the biomarker distribution that governs prognosis, and the beneficial effects of treatment may be mediated through other distributional characteristics. An example is blood pressure variability. Studies have shown that blood pressure variability is prognostic for cardiovascular outcomes and clinical trials assessing interventions that decrease blood pressure variability have been recommended [3]. Such trials would assess the effect of treatment on the variance of a biomarker distribution, for which distributional regression is well suited.

### Development of regression modelling

In the case of a two-armed trial with continuous outcome *y*, the classical linear model specifies

$${y}_i\left|{t}_i,{x}_i\sim \mathcal{N}\left({\mu}_i,{\sigma}^2\right)\right.\kern2em independently,\kern0.5em for\kern0.5em i=1, \ldots ,n$$

$$\mathrm{E}\left({y}_i\left|{t}_i,{x}_i\right.\right)={\mu}_i={\beta}_0+{\beta}_t{t}_i+{\beta}_1{x}_{i1}+\cdots +{\beta}_p{x}_{ip}$$

where *t*_*i*_ is an indicator variable for treatment, *β*_*t*_ is the treatment effect and **x**_*i*_ = (*x*_*i*1_, …, *x*_*ip*_)^⊤^ is a covariate vector whose elements may be continuous or binary. While taking logarithms or other transformations of *y* and/or some of the covariates is sometimes helpful for satisfying the normality and linearity assumptions, the restrictive model assumptions of (conditional) normality, homoscedasticity and linearity are in practice frequently not met. Nevertheless this model remains popular for analysis.

Relaxation of the classical linear model assumptions started with the GLM: “Theoretical and applied statistics were both convulsed by the publication of the GLM paper by Nelder & Wedderburn (1972).” [4]. The revolutionary aspects of the GLM were the extension of the choice of response distribution to any member of the exponential family of distributions, which includes the normal, Poisson, binomial, Gamma, inverse Gaussian and Tweedie distributions; inclusion of a link function *g*(·) in the model specification for *μ*_*i*_:

1 $$g\left({\mu}_i\right)={\beta}_0+{\beta}_t{t}_i+{\beta}_1{x}_{i1}+\cdots +{\beta}_p{x}_{ip}$$

where *g*(·) is any monotonic differentiable function; and an algorithm for the computation of the maximum likelihood estimates which was computationally feasible within computing constraints at the time. Since then development of the regression framework has included broadening the linear predictor to include smooth terms (defined below); the extension of allowed response distributions to any parametric distribution which is twice differentiable and computable; and the ability to specify model equations similar to (1) for *any* distribution parameter, not just the mean. For example, a normal linear model which displays variance which is dependent on the treatment and/or covariate(s) (i.e. heteroscedasticity) is accommodated:

$${y}_i\left|{t}_i,{x}_i\sim\mathcal{N}\left({\mu}_i,{\sigma}_i^2\right)\right.\kern1.5em independently,\kern0.5em for\kern0.5em i=1, \ldots ,n$$

$${\mu}_i={\beta}_0^{\mu }+{\beta}_t^{\mu }{t}_i+{\beta}_1^{\mu }{x}_{i1}+\cdots +{\beta}_p^{\mu }{x}_{ip}$$

$$\log \left({\sigma}_i^2\right)={\beta}_o^{\sigma }+{\beta}_t^{\sigma }{t}_i+{\beta}_1^{\sigma }{x}_{i1}+\cdots +{\beta}_p^{\sigma }{x}_{ip}$$

The superscripts on the β's denote the parameter to which the coefficient corresponds. So, for example, ${\beta}_t^{\mu }$ is the treament effect on *μ*, while ${\beta}_t^{\sigma }$ is the treatment effect on *σ* ^2^. The logarithmic link for *σ* ^2^ guarantees positivity of parameter estimates ${\hat{\sigma}}_i^2$; other link functions may also be used [5]. Note that not all of *x*_1_, …, *x*_*p*_ and *t* need be present in the model equations for both *μ* and *σ* ^2^.

GAMLSS modelling is implemented in the R package **gamlss** [6, 7], in which over one hundred response distributions are currently available. These enable modelling of the following types of outcome variable, without the need for data transformation:

- continuous outcomes (positive or negative);
- continuous, non-negative outcomes;
- counts (bounded or unbounded);
- proportions (continuous, on the interval zero to one);
- any of the above with inflated probabilities at zero, and/or one in the case of proportions.

Parameter estimation in **gamlss** is performed using the method of maximum likelihood, resulting in the coefficient estimates having the usual asymptotic properties of maximum likelihood estimates, viz. normality, unbiasedness, consistency and efficiency. Bayesian estimation for GAMLSS models, which we do not cover in this paper, is available in the R package **bamlss** [8].

In this paper we focus on the modelling of continuous outcomes.

### Model terms

The model equations given above are of the following (linear) form, for generic parameter *θ*, and which we denote as ${\eta}_i^{\theta }$:

$$g\left({\theta}_i\right)={\eta}_i^{\theta }={\beta}_0^{\theta }+{\beta}_t^{\theta }{t}_i+{\beta}_1^{\theta }{x}_{i1}+\cdots +{\beta}_p^{\theta }{x}_{ip}$$

Should the effect of a covariate *x*_*j*_ on ${\eta}_i^{\theta }$ be nonlinear, we can, in order of preference:

- find a nonlinear transformation of *x*_*j*_ that captures the relationship; or
- use a “smooth function” of *x*_*j*_, discussed below; or
- categorize *x*_*j*_ and enter it into the model as a factor.

A nonlinear transformation, if justified by the data (and possibly the underlying science), is arguably the best approach to a nonlinear relationship. However, should an appropriate transformation not be obvious (as is frequently the case), smooth functions are an excellent alternative. They were introduced into regression modelling as spline terms in Generalized Additive Models, by [9]. (A good recent reference is [10].) The linear predictor becomes

$${\eta}_i^{\theta }={\beta}_0^{\theta }+{\beta}_t^{\theta }{t}_i+{s}_1^{\theta}\left({x}_{i1}\right)+\cdots +{s}_p^{\theta}\left({x}_{ip}\right)$$

where the ${s}_j^{\theta}\left({x}_{ij}\right)$ can be linear terms (${\beta}_j^{\theta }{x}_{ij}$) or smooth functions. There are a few alternatives available for smooth functions, most notably splines, lowess and fractional polynomials. Mathematically and computationally, spline terms are fairly easily handled as they are composed of a series of linear terms (or *basis functions*), so the model retains its linear structure. There is a tradeoff between complexity (curve “too wiggly,” too sensitive to local variations) and simplicity (curve “too smooth,” not sensitive to important variations), which is resolved in model estimation by using penalties for curve complexity.

### Random effects

Clinical trials often involve dependence structures induced by design features such as clustered randomization, longitudinal and repeated measurement on individuals, multiple endpoints, crossover of treatments and many others. Distributional regression using the GAMLSS model has the capacity to introduce random effects into the linear predictor for any or all of the parameters being modelled. Such random effects models can capture the effect of these dependence structures on parameters other than the mean, using mixed GAMLSS models that are analogous to standard mixed models for correlated data. The software and computational tools described below have the capacity to incorporate these random effects in a straightforward manner, making the flexible distributional regression framework available in contexts where dependence must be incorporated into the model.

### The GAMLSS model

The full GAMLSS model for a two-armed trial is

2 $${y}_i\sim \mathcal{D}\left({\mu}_i,{\sigma}_i,{\nu}_i,{\tau}_i\right)\kern1.5em independently,\kern0.5em for\kern0.5em i=1, \ldots ,n$$

$${g}_1\left({\mu}_i\right)={\beta}_0^{\mu }+{\beta}_t^{\mu }{t}_i+{s}_1^{\mu}\left({x}_{i1}\right)+\cdots +{s}_p^{\mu}\left({x}_{ip}\right)$$

$${g}_2\left({\sigma}_i\right)={\beta}_0^{\sigma }+{\beta}_t^{\sigma }{t}_i+{s}_1^{\sigma}\left({x}_{i1}\right)+\cdots +{s}_p^{\sigma}\left({x}_{ip}\right)$$

$${g}_3\left({\nu}_i\right)={\beta}_0^{\nu }+{\beta}_t^{\nu }{t}_i+{s}_1^v\left({x}_{i1}\right)+\cdots +{s}_p^v\left({x}_{ip}\right)$$

$${g}_4\left({\tau}_i\right)={\beta}_0^{\tau }+{\beta}_t^{\tau }{t}_i+{s}_1^{\tau}\left({x}_{i1}\right)+\cdots +{s}_p^{\tau}\left({x}_{ip}\right)$$

where $\mathcal{D}\left(\cdot \right)$ is a parametric distribution with computable first and second derivatives and having up to four parameters *μ*, *σ*, *ν* and *τ*; *g*_*k*_(·) is the link function for the *k*th parameter, which is monotonic and differentiable; and the functions *s*_*j*_(·) represent a variety of different effects: linear or nonlinear effects of continuous covariates, smooth terms (usually implemented mathematically as splines), spatial effects, or random effects.

Note that the response distribution $\mathcal{D}$ may have one, two, three or four distribution parameters; of these, not all need to be modelled with covariates; and the sets of covariates in the models for distribution parameters may be the same, disjoint or overlapping.

## Methods

### Software

Estimation of GAMLSS models is implemented using penalized maximum likelihood, or Bayesian estimation, in the R packages **gamlss** [7] and **bamlss** [8] respectively. We have used **gamlss** for estimation throughout.

### LIPID trial

The Long-Term Intervention with Pravastatin in Ischaemic Disease (LIPID) trial is a double-blind, randomized controlled trial of patients with stable coronary heart disease and a broad range of cholesterol levels. The trial compared the effects of pravastatin with those of placebo in 9014 patients over a mean follow-up of 6.1 years. Both treatment arms received advice on a cholesterol-lowering diet. The primary outcome of the trial was mortality from coronary heart disease, and they found a relative risk reduction of 24% (95% CI, 12–35, *p* < 0.001). The trial also found lower overall mortality and lower incidence of all cardiovascular outcomes in patients treated with pravastatin [11].

### Description of biomarker MR-proADM

The LIPID trial has also published data on the long term effects of treatment with pravastatin [12] and the analysis of eight biomarkers used in coronary heart disease [13]. One of the eight biomarkers measured in the LIPID study is plasma midregional pro-adrenomedullin (MR-proADM), which is a surrogate marker for adrenomedullin release. Adrenomedullin acts as a vasodilator and has important roles in microcirculation and endothelial dysfunction. Lower baseline and reductions in the change of MR-proADM have been associated with decreased risk of major clinical events, even after adjustment for other important biomarkers such as B-type natriuretic peptide (BNP).

## Results

### MR-proADM

**Fig. 1:**
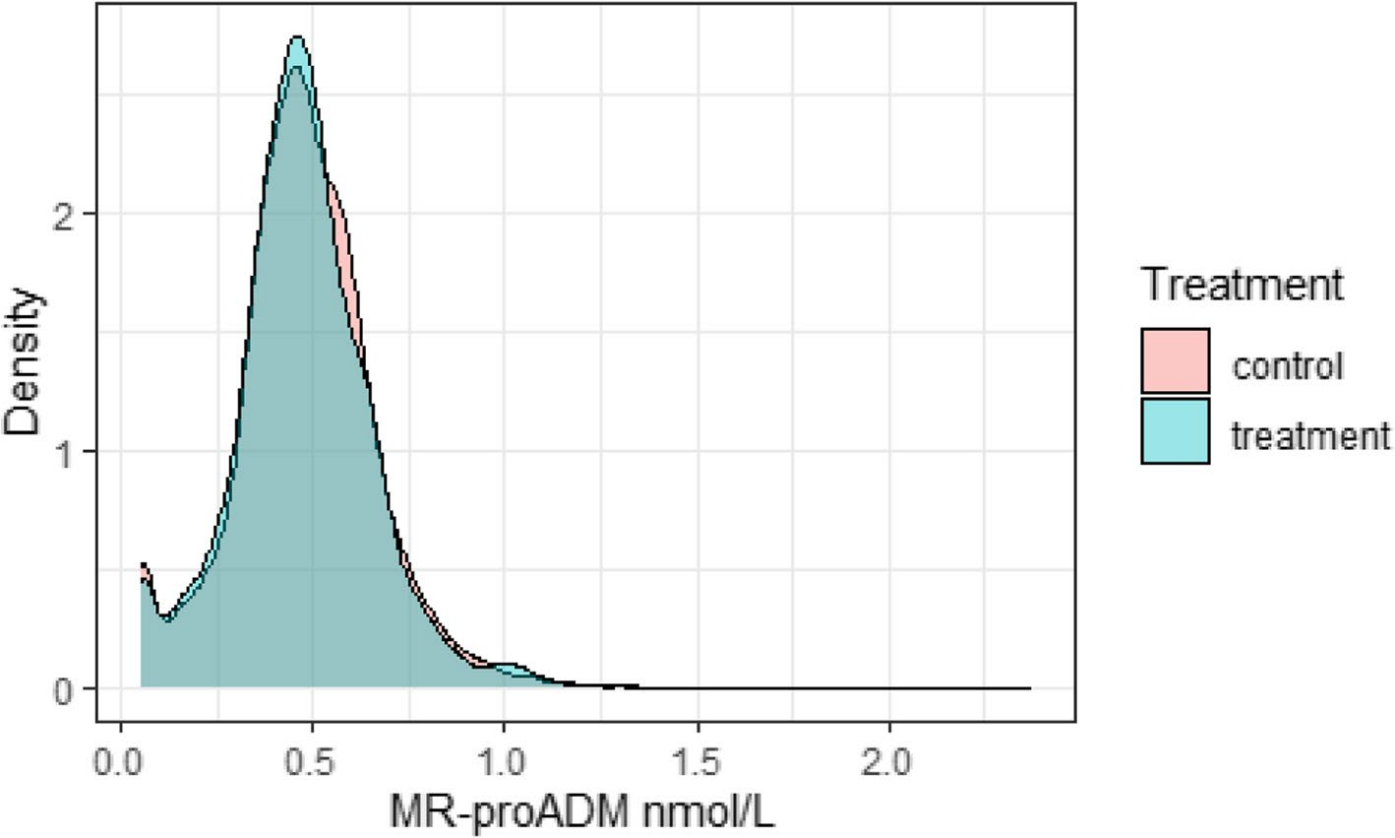
Density plots of MR-proADM at month 12, treatment and control groups. The densities are kernel density estimates, computed using the R package ggplot2

The detection limit of MR-proADM is 0.05 nmol/L, so it is assumed to be left-censored at this point. Figure 1 shows density plots of MR-proADM at 12 months; there appears to be a small beneficial treatment effect.

### GAMLSS analysis

GAMLSS has the functionality for the creation of censored versions of any of its distributions; in this case, we found the Box-Cox t (BCT) distribution [14], left-censored at 0.05, to provide the best fit, according to the AIC criterion. (This was found using the **gamlss** function *fitDist*, which performs a search of all appropriate distributions (in this case, continuous distributions on the positive real line), and ranks them in order of Akaike information criterion (AIC)). The BCT is a four-parameter, non-negative continuous distribution, with *μ* being the median (approximately), *σ* the (approximate) coefficient of variation, *ν* a skewness parameter and *τ* a kurtosis parameter.

We contrast the left-censored normal and left-censored BCT regression models. For comparability with the normal model, we initially only fit the treatment and covariate effects to the parameter *μ* of the BCT (“reduced model”).

1. Reduced left-censored normal model:

$${y}_i\left|{t}_i,{x}_i\sim N\left({\mu}_i,{\sigma}^2,0.05\right)\right.$$

2. Reduced left-censored BCT model:

$${y}_i\left|{t}_i\right.,{x}_i\sim BCT\left({\mu}_i,\sigma, v,\tau, 0.05\right)$$

where *y*_*i*_ is the level of MR-proADM activity at month 12 and, for both models, the last argument (0.05) signifies the left-censoring cutoff. The model equation for *μ* is

3 $$\log \left({\mu}_i\right)={\beta}_0^{\mu }+{\beta}_t^{\mu }{t}_i+{\beta}_1^{\mu}\log \left({x}_i\right),$$

where *t*_*i*_ is an indicator for treatment and *x*_*i*_ is baseline MR-proADM activity. The logarithmic link for *μ* ensures non-negativity; the assumption of proportionality implicit in (3) is shown to be satisfied in scatterplots of the logarithms of month 12 and baseline biomarker activity, which are strongly linear (Supplementary material).

**Fig. 2:**
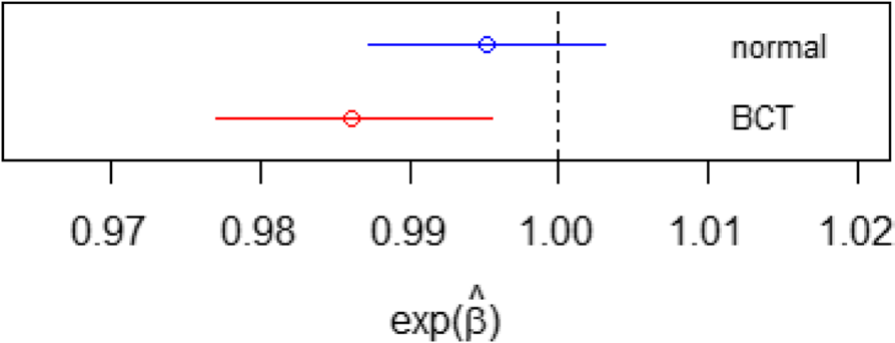
Estimated treatment effect and 95% confidence intervals on μ, reduced BCT and normal models for MR-proADM

We use the AIC as criterion for selection of the covariates in the “extended models” for both distributions. Covariates available for analysis were sex and baseline MR-proADM activity. Parameter estimates for the four models are shown in Table 1, and a plot of the treatment effect on the parameter *μ* as estimated in the reduced models, with 95% confidence intervals, in Fig. 2. (Because of the log link, we display $\exp \left({\hat{\beta}}_{\mathrm{t}}^{\mu}\right)$, with the value 1 being consistent with no treatment effect.) Note that the BCT model concludes a significant beneficial treatment effect on the median, whereas the normal model does not. The extended models both include sex as a predictor for *σ*, and the BCT extended model also includes a treatment effect for *σ* and *ν*, and baseline MR-proADM in the models for *σ*, *ν* and *τ*.

**Table 1:** Parameter estimates under left-censored reduced and extended BCT and normal models

		Reduced model						Extended model					
		BCT			NO			BCT			NO		
Parameter	Coefficient	Estimate	SE	p	Estimate	SE	p	Estimate	SE	p	Estimate	SE	p
μ	(Intercept)	−0.129	0.007	< 0.001	0.668	0.005	< 0.001	− 0.088	0.006	< 0.001	0.745	0.008	< 0.001
μ	baseline	0.813	0.009	< 0.001	0.236	0.004	< 0.001	0.879	0.008	< 0.001	0.319	0.008	< 0.001
μ	sex										−0.017	0.006	0.003
μ	treatment	−0.014	0.005	0.004	−0.005	0.004	0.224	−0.018	0.005	< 0.001	−0.005	0.004	0.166
σ	(Intercept)	−1.959	0.019	< 0.001	− 1.818	0.009	< 0.001	−2.796	0.050	< 0.001	−1.890	0.025	< 0.001
σ	baseline							−1.426	0.039	< 0.001	−0.230	0.017	< 0.001
σ	sex							−0.158	0.037	< 0.001	−0.154	0.024	< 0.001
σ	treatment							0.073	0.030	0.014			
ν	(Intercept)	0.426	0.069	< 0.001				1.109	0.105	< 0.001			
ν	baseline							0.153	0.045	0.001			
ν	treatment							0.124	0.076	0.101			
τ	(Intercept)	0.287	0.007	< 0.001				−0.029	0.006	< 0.001			
τ	baseline							−1.184	0.008	< 0.001

**Fig. 3:**
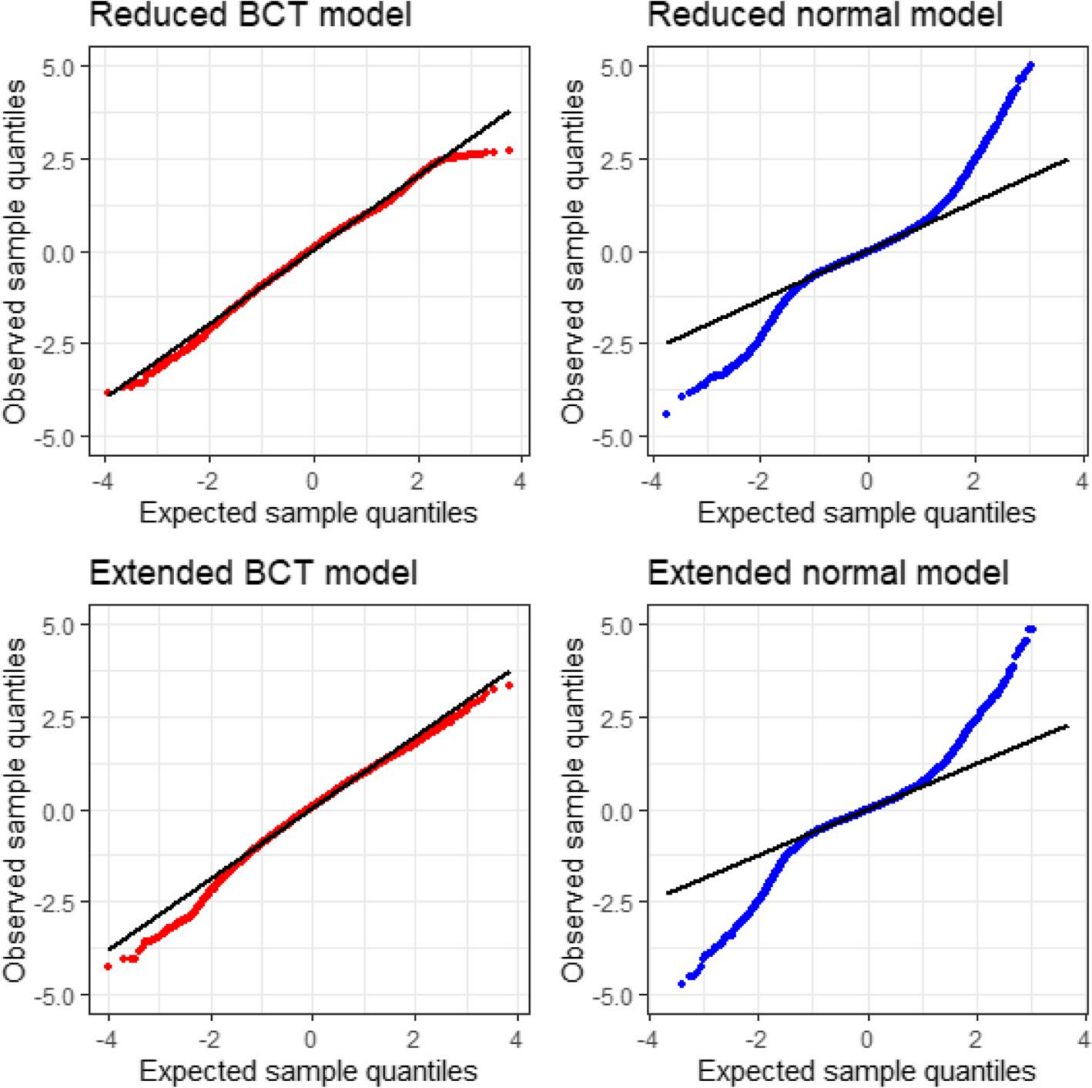
Normal plots of the normalized quantile residuals, reduced BCT and normal models for MR-proADM

Quantile-quantile plots of the normalized quantile residuals are given in Fig. 3; the BCT models provide good fits, whereas the normal model fits are poor. A comparison of the four models using the AIC is given in Table 2. Both BCT models are preferred to the normal models, and of these the extended BCT model is preferred.

**Table 2:** Comparison of models for MR-proADM using the AIC

Model	df	AIC
extended BCT	12	− 8352
reduced BCT	6	− 7147
extended normal	7	− 4869
reduced normal	4	− 4648

**Fig. 4:**
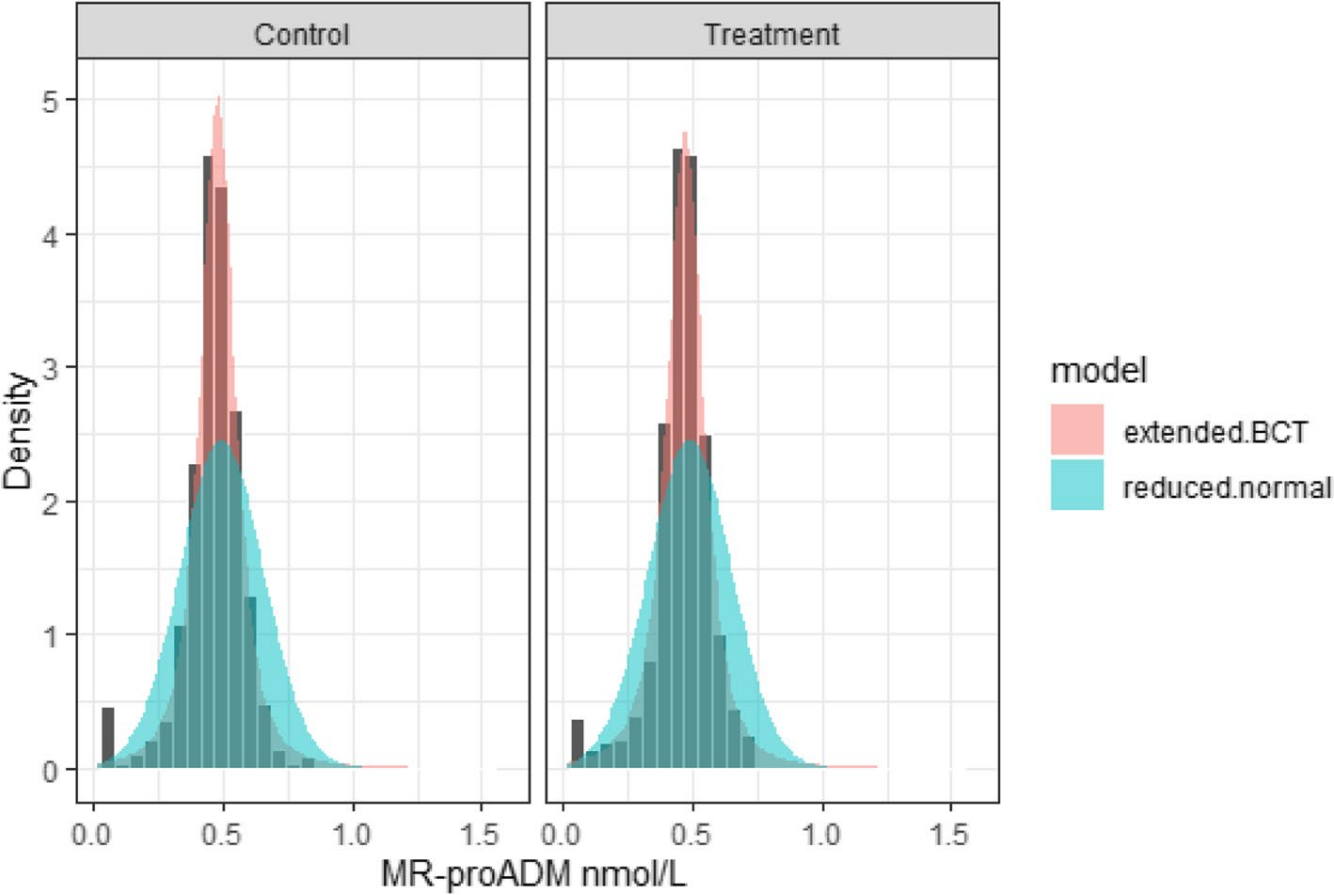
Fitted PDFs of the reduced normal and extended BCT models, with baseline MR-proADM at its median and male gender, superimposed on the histogram of the response data in the neighbourhood of the median of baseline MR-proADM and male gender

Figure 4 shows fitted probability density functions (PDFs) of the reduced normal and extended BCT models, with baseline MR-proADM activity at its median and male gender, superimposed on the histogram of the response data in the neighbourhood of the median of baseline MR-proADM and male gender. It can be seen that the BCT model successfully captures the sharp peak in the observed data, whereas the normal model is unable to model this feature and compensates with greater variance than is observed.

### Systolic blood pressure

**Fig. 5:**
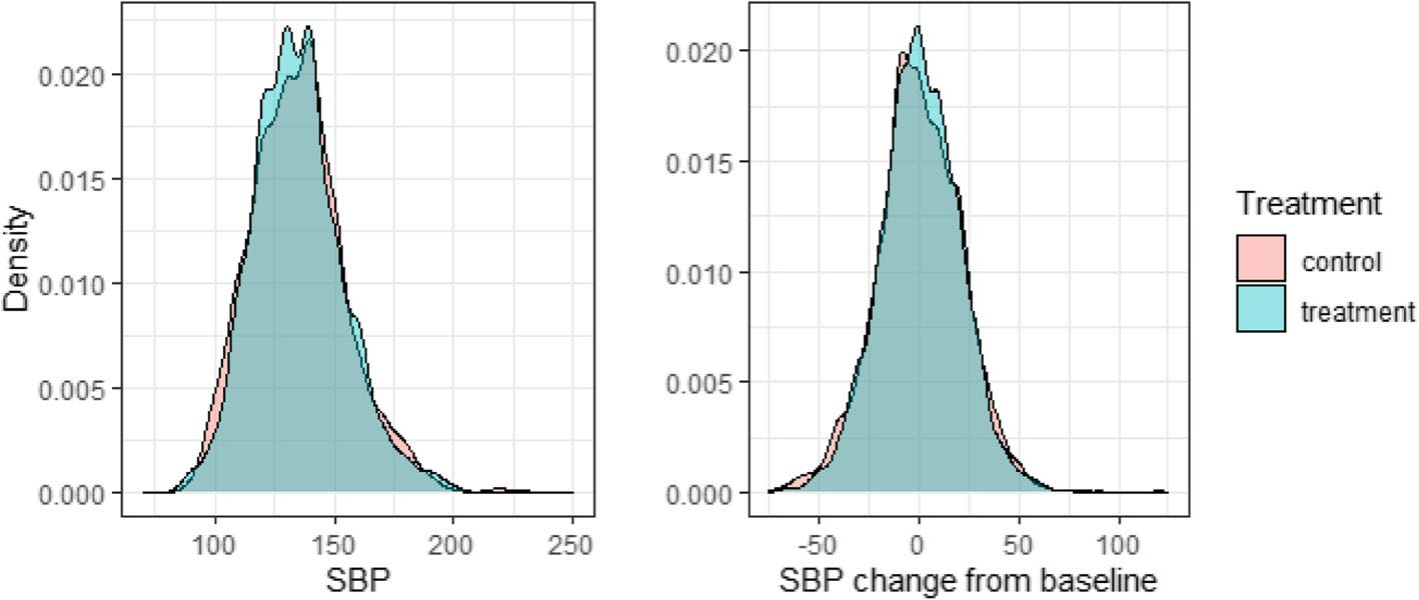
Density plots of SBP at 72 months (left) and SBP change from baseline at 72 months (right), by treatment group. The densities are kernel density estimates, computed using the R package ggplot2

SBP is the pressure in arteries during the contraction of the heart muscle, and is a major risk factor for cardiovascular disease in people over 50 years old. SBP generally increases with age, as blood vessels stiffen and plaque builds up over time. SBP was measured 12-monthly from baseline in the LIPID trial. We initially analyse change from baseline at 72 months, and subsequently change from baseline at 48 and 72 months, as a longitudinal model. Density plots of SBP at 72 months and SBP change from baseline at 72 months, by treatment group, are shown in Fig. 5.

### Models for change in SBP at 72 months

The Johnson’s *S*_*u*_ (JSU) distribution [14] was found to provide the best fit to change in SBP at 72 months. The JSU is a four-parameter continuous distribution, with parameters *μ* (the mean), *σ* (the standard deviation), *ν* (skewness parameter) and *τ* (kurtosis parameter). The JSU looks somewhat similar to the normal distribution when *ν* = 0, but is capable of skewness and more kurtosis (‘more peaked’) than the normal. Plots of the JSU pdf are shown in the Supplementary Material.

Model selection using the AIC, with the JSU as response distribution, resulted in the extended JSU model given in Table 3. Reduced JSU and normal models, and the extended normal model, are also shown.

**Table 3:** Parameter estimates under reduced and extended JSU and normal models

		Reduced model						Extended model					
		JSU			NO			JSU			NO		
Parameter	Coefficient	Estimate	SE	p	Estimate	SE	p	Estimate	SE	p	Estimate	SE	p
μ	(Intercept)	82.142	2.456	< 0.001	80.322	2.550	< 0.001	79.722	2.576	< 0.001	79.889	2.560	< 0.001
μ	baseline	−0.609	0.018	< 0.001	−0.594	0.019	< 0.001	− 0.589	0.019	< 0.001	− 0.590	0.019	< 0.001
μ	treatment	0.514	0.676	0.447	0.101	0.706	0.886	0.042	0.694	0.952	0.041	0.704	0.954
σ	(Intercept)	2.867	0.017	< 0.001	2.868	0.014	< 0.001	2.473	0.125	< 0.001	2.519	0.100	< 0.001
σ	baseline							0.003	0.001	< 0.001	0.003	0.001	< 0.001
σ	treatment							−0.067	0.031	0.030	−0.073	0.028	0.011
ν	(Intercept)	1.259	0.353	< 0.001				4.018	2.178	0.065			
ν	baseline							−0.018	0.014	0.195			
τ	(Intercept)	1.057	2.456	< 0.001				1.160	2.576	< 0.001			

In the extended JSU model, the treatment effect on the mean *μ* is non-significant (*p* = 0.952). However treatment is significant for the standard deviation *σ* (*p* = 0.030), in the direction of reduced variability for the active treatment. The extended normal model also finds a significant treatment effect on *σ*, and no significant treatment effect on *μ*. Parameter estimates for *μ* and *σ* are similar for the extended JSU and normal models; however, for the reduced models they are quite different.

**Fig. 6:**
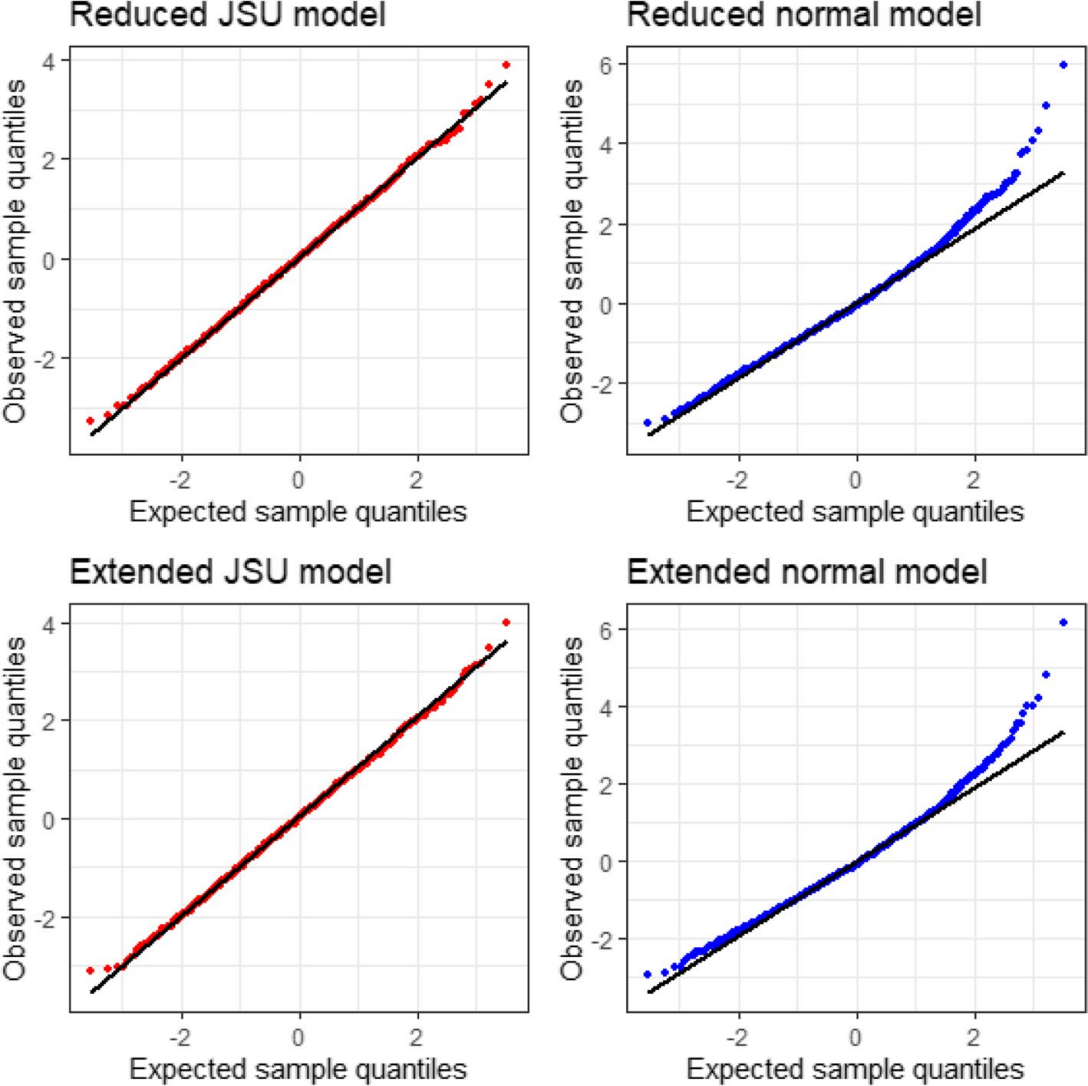
Normal plots of the normalized quantile residuals, reduced and extended JSU and normal models for change in SBP at 72 months

Normal plots of the normalized quantile residuals of the four models are shown in Fig. 6. As for the previous example, the normal models display a lack of fit, particularly in the upper tail, whereas the JSU models fit particularly well.

### Longitudinal analysis of change in SBP at months 48 and 72

It may be of interest to model SBP trajectories over the follow-up period. In this case we are dealing with longitudinal observations, in which each participant has repeated observations which are assumed to be correlated. Modelling of correlated outcomes is achieved using one of two approaches: random effects, or generalized estimating equations (GEE). In the distributional regression framework, random effects methodology is well developed and we illustrate their application in this example. (Although in principle GEE models could also be incorporated in the distributional regression framework, this methodology does not appear to have been developed.)

To facilitate comparison with the extended JSU model for change in SBP at 72 months, we have implemented a random effects model for change in SBP at 48 and 72 months, using the same set of covariates as the former model as well as an effect for month (72 vs 48) for *μ* and *σ*. A random intercept for subject, for the parameter *μ*, has been included in order to model within-subject correlation. Estimates are given in Table 4: as for the model for month 72, there is a significant treatment effect for *σ* (*p* = 0.001) but not for *μ* (*p* = 0.381). The estimated multiplicative effect of treatment on *σ* is exp(− 0.048) = 0.953, i.e. a decrease in standard deviation of 4.7%. There is also a significant effect of month (72 vs 48) on both *μ* (*p* = 0.006) and *σ* (*p* = 0.012), in the direction of increased *μ* and *σ* at month 72 compared with month 48.

**Table 4:** Parameter estimates for longitudinal JSU model for change in SBP at 48 and 72 months

Parameter	Coefficient	Estimate	SE	*p* value
μ	(Intercept)	79.238	0.830	< 0.001
μ	baseline SBP	−0.592	0.006	< 0.001
μ	month 72	0.747	0.271	0.006
μ	treatment	0.200	0.229	0.381
μ	random effect	10.712	0.116	< 0.001
σ	(Intercept)	1.862	0.051	< 0.001
σ	baseline SBP	0.005	0.000	< 0.001
σ	month 72	0.042	0.016	0.012
σ	treatment	−0.048	0.014	0.001
ν	(Intercept)	5.354	0.617	< 0.001
ν	baseline SBP	−0.026	0.004	< 0.001
τ	(Intercept)	0.950	0.020	< 0.001

**Fig. 7:**
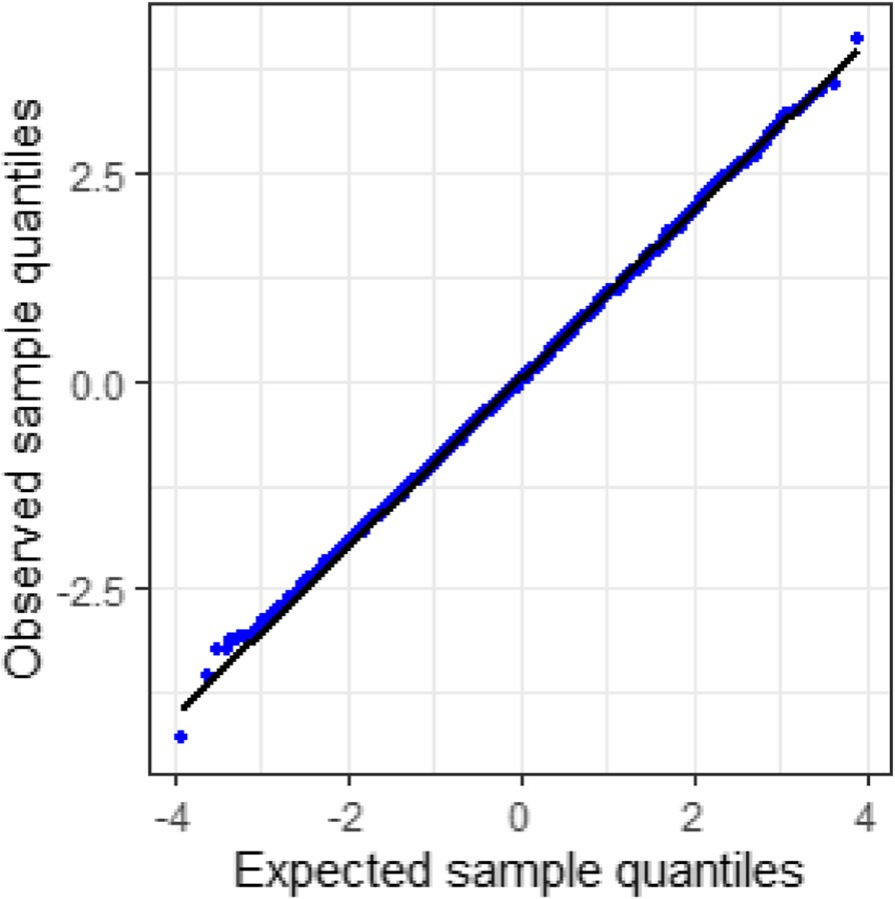
Normal plot of the normalized quantile residuals, longitudinal JSU model for change in SBP at 48 and 72 months

The normal plot of the normalized quantile residuals of the longitudinal model (Fig. 7) shows a well-fitting model.

### Simulation study

We have demonstrated the usefulness of distributional regression in the analysis of clinical trials. In order to provide evidence that it delivers reliable estimates of treatment effect that are superior to those based on the normal linear model, we have conducted a simulation study, based on the MR-proADM biomarker in the LIPID study. The simulation used the treatment allocations and baseline MR-proADM of the *n* = 6, 539 participants who had no missing values for MR-proADM at month 12. Baseline MR-proADM was included in all models for *μ*, and in models for *σ* in the case of extended models.

In the first simulation, we used the extended normal model for 12-month MR-proADM (see Table 1) as generating model. Using fitted means ${\hat{\mu}}_i$ and variances ${\hat{\sigma}}_i^2$, we simulated 500 samples with normally distributed responses from $\mathcal{N}\left({\hat{\mu}}_i,{\hat{\sigma}}_i^2\right)$, for *i* = 1, …, *n*. For each sample (*j* = 1, …, 500), we

- chose the best distribution using the AIC as selection criterion. We chose from the following distributions: normal, JSU, BCT and skew normal;
- using the chosen distribution and a treatment effect specified on the mean only in the case of the normal (i.e. the reduced normal model), and *μ* and *σ* for distributions other than the normal, estimated the treatment effect on *μ* and its standard error.

In the second simulation, we used the extended BCT model for 12-month MR-proADM (see Table 1) as generating model. Using fitted values ${\hat{\mu}}_i$, ${\hat{\sigma}}_i$, $\hat{\nu}$ and $\hat{\tau}$, we simulated 500 samples with responses from $\mathrm{BCT}\left({\hat{\mu}}_i,{\hat{\sigma}}_i,\hat{\nu},\hat{\tau}\right)$, for *i* = 1, …, *n*. For each sample (*j* = 1, …, 500), we

- chose the best distribution as above,
- using the chosen distribution and a treatment effect specified on *μ* and *σ*, estimated the treatment effect on *μ* and its standard error, and
- compared the estimates with those obtained from a reduced normal model.

Table 5 shows the results of the response distribution selection for both simulations. In the case of the normal generating model, the normal was chosen in 86% of samples, and the JSU in the remaining 14%. For the BCT generating model, the BCT was chosen in 100% of samples.

**Table 5:** Chosen distributions in Normal and BCT simulations

	Generating distribution	
	Normal^1^	BCT^1^
**Chosen distribution**		
BCT	0 (0%)	500 (100%)
JSU	72 (14%)	0 (0%)
Normal	428 (86%)	0 (0%)
Skew normal	0 (0%)	0 (0%)

^1^n (%)

Table 6 shows the results of the first simulation, in which the generating model is the normal and the chosen distributions were the normal and JSU. As the parameter *μ* is the mean for both the normal and JSU distributions, parameter estimates for the model for *μ* are comparable. For the samples for which the JSU distribution was chosen, all of the 95% confidence intervals for the treatment effect on the mean included the true (simulated) effect. For the samples for which the normal distribution was chosen, 96% of the 95% confidence intervals cover the true (simulated) effect. The average standard error of the treatment effect on the mean was, in both cases, 0.004.

**Table 6:** Results of simulation: Normal generating distribution, treatment effect on the mean

	JSU, *N* = 72^1^	NO, *N* = 428^1^
Coverage rate of 95% CI for β	72 (100%)	409 (96%)
Average standard error	0.004	0.004

^1^n (%); Mean

These results are reassuring that, when the underlying model is normal, distributional regression does not introduce any disadvantage. In the 14% of cases in which the normal was not the chosen distribution, the JSU was chosen. This distribution is similar to the normal, with more flexibility provided by its four parameters. The coverage rate for ${\beta}_t^{\mu }$ under JSU estimation was better than the normal; and standard errors provided by the two estimating models were the same (to three decimal places).

Results of the second simulation, in which the generating model is the BCT, are shown in Table 7. In all cases the BCT was the chosen distribution; we compare this with estimation under the normal linear (reduced) model. The parameter *μ* is the mean in case of the normal estimating model, and the (approximate) median in the case of the BCT estimating model. While these are both location parameters, they are not the same and as a result their parameter estimates are not directly comparable. Instead we compare the rejection rates of the hypothesis of no treatment effect on *μ*:

$${H}_{01}:{\beta}_t^{\mu }=0$$

**Table 7:** Rejection rates for specified hypotheses, under the BCT generating model

Estimating model	Hypothesis	Rejection rate
Extended BCT	H_01_	96.2%
Extended BCT	H_02_	94.8%
Reduced normal	H_01_	26.8%

for the normal and BCT estimating models, using the Wald test. Standard errors for coefficient estimates, based on the inverse of the observed information matrix, are available from **gamlss** output and were used to generate the Wald statistics. For the BCT estimating model we also tested the hypothesis of no overall treatment effect on *μ* and *σ*:

$$H_{02}:\left(\begin{array}{l} \beta_{t}^{\mu} \\ \beta_{t}^{\sigma} \end{array}\right)=\left(\begin{array}{l} 0 \\ 0 \end{array}\right)\ ,$$

using the likelihood ratio test statistic with null distribution the chi-square with two degrees of freedom. Results are shown in Table 7.

While estimation under the extended BCT model produces acceptably high rejection rates, the rejection rate for ${\beta}_t^{\mu }$ of the reduced normal model is poor. This confirms the result observed in Table 1 and Fig. 2, in which treatment effect on the parameter *μ* of the BCT model was significant, but the corresponding estimate under the normal model failed to reach significance.

## Discussion and conclusions

We have demonstrated some of the richness of the distributional regression modelling framework, and its capability of accurately modelling continuous outcomes with features different from the normal distribution.^1^ Since its first publication, GAMLSS has gained increasing popularity in a wide variety of fields of application [15]. Distributional regression offers not only a very wide choice of response distribution, but also the ability to model parameters other than the mean; random effects to accommodate clustered observations; smooth terms for modelling nonlinear effects; and zero inflation and censoring. Particularly noteworthy in the context of clinical trials, is the ability to model a treatment effect on a feature of the response distribution other than the mean, for example the variance. Traditionally the analysis of trials is focused on estimation of the treatment effect, implicitly assumed to be on the mean. In fact a treatment effect on the variance of the response may be of importance, for example for the outcome blood pressure, and conventional normal modelling does not have the capability to detect this. This points to a need for a rethink of the interpretation of the notion of “treatment effect” when treatment is included in distribution parameters other than the mean (or median).

In our two examples, under the normality assumption the lack of fit was not improved by the addition of a model for *σ*; however the use of more appropriate response distributions resulted in well-fitting models. Where the normal linear model is used in situations in which model assumptions are not met, estimates of treatment effect based on the misspecified model will be biased [16] show that, under fairly general conditions, a large class of regression models (Gaussian, binomial, Poisson) yield asymptotically correct Type I errors for hypotheses on treatment effect, even when the models are incorrectly specified. This robustness to violation of model assumptions suggests that, if the hypothesis concerning treatment effect is their only goal, analysts need not be too concerned about lack of model fit as long as the conditions are met. This has not been our experience; we have found in our applications and via simulation, that use of the normal model when the underlying generating model is non-normal and heteroscedastic, can lead to poor power for the detection of a treatment effect. Consequently if interest lies in accurate detection and estimation of treatment effect on the mean, or distributional features other than the mean, use of distributional regression modelling will yield superior estimates and is strongly recommended.

## Supplementary Information

**Additional file 1.** Supplementary material: MR-proADM scatterplot.

**Additional file 2.** LIPID site and Ethics committee.

**Additional file 3.** Supplementary material: Plot of JSU distribution.

## Data Availability

The LIPID data set is held by the National Health and Medical Research Council Clinical Trials Centre, University of Sydney. Complete individual patient data have been provided to other research groups for meta-analysis. Proposals for analyses or collaborative studies by researchers are welcome and should be submitted to the corresponding author. The R code used to analyse the data is available from the corresponding author on request.

## Abbreviations

AIC: Akaike information criterion
BCT: Box-Cox t distribution
GAMLSS: Generalized Additive Models for Location, Scale and Shape
GEE: generalized estimating equations
GLM: generalized linear model
JSU: Johnson’s Su distribution
LIPID: Long-Term Intervention with Pravastatin in Ischaemic Disease
MR-proADM: midregional pro-adrenomedullin
PDF: probability density function
SBP: systolic blood pressure
SE: standard error

## Footnotes

1 Discrete outcomes may also be modelled in this framework; we have not discussed these in this paper.
