# Supplementary material for: Distributional regression in clinical trials: treatment effects on parameters other than the mean
Source: BMC Med Res Methodol. 2022 Feb 27;22:56. doi: 10.1186/s12874-022-01534-8 (PMC8883706; doi:10.1186/s12874-022-01534-8)
Supplement: Supplementary file 1 — Additional file 1. Supplementary material: MR-proADM scatterplot. [file 12874_2022_1534_MOESM1_ESM.pdf]

Supplementary material

MR-proADM scatterplot

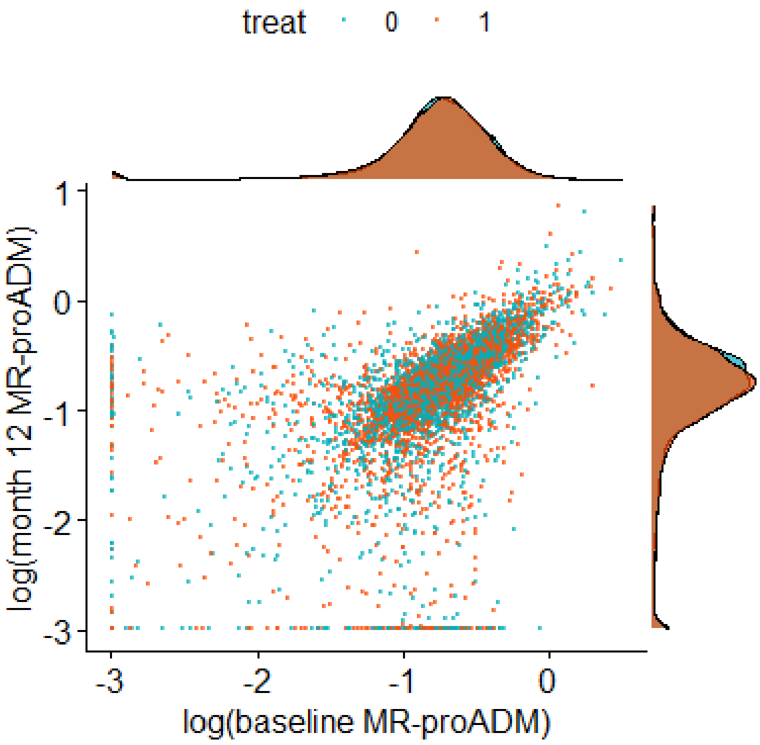

Figure 1: Scatterplot of log(month 12 MR-proADM) and log(baseline MR-proADM), which shows a strong linear relationship. Kernel density estimates of the baseline and month 12 levels are shown on the outer axes.
