# Supplementary material for: Distributional regression in clinical trials: treatment effects on parameters other than the mean
Source: BMC Med Res Methodol. 2022 Feb 27;22:56. doi: 10.1186/s12874-022-01534-8 (PMC8883706; doi:10.1186/s12874-022-01534-8)
Supplement: Supplementary file 2 — Additional file 2. LIPID site and Ethics committee. [file 12874_2022_1534_MOESM2_ESM.pdf]

LIPID Site	Ethics Committee
The Canberra Hospital	ACT Department of Health and Community Care
Royal Prince Alfred Hospital, Sydney	Central Sydney Area Health Service
Royal North Shore Hospital,Sydney	Royal North Shore Hospital
Concord Repatriation General Hospital,Sydney	CSAHS Ethics Review Committee – CRGH Zone
St Vincents Hospital, Sydney	St Vincents Hospital
Dr K Wee (private practice), Kempsey	Mid North Coast Area Health Service
St George Hospital, Sydney	South Eastern Sydney Area Health Service, Southern
Bowral & District Hospital, NSW	South Eastern Sydney Area Health Service
Canterbury Hospital, Sydney	Central Sydney Area Health Service
Prince Henry Hospital, Sydney	South Eastern Sydney Area Health Service
Dr J England (private practice),Katoomba	Wentworth Area Health Service
Dr J Waites (private practice), Coffs Harbour	Flinders Medical Centre
Gosford District Hospital, NSW	Central Coast Area Health Service
Hornsby District Hospital, Sydney	Hornsby, Ryde and Macquarie Hospitals Ethics Committee
Dr A Neaverson (private practice), Sydney	Central Sydney Area Health Service
John Hunter Hospital, Newcastle	Hunter Area Research Ethics Committee
Dr B Cuthbert (private practice) Tweed Heads	John Flynn Hospital Ethics Committee
Nepean Hospital, Sydney	Wentworth Area Health Service
Wollongong Hospital, NSW	University of Wollongong Human Research Ethics Committee
Albury Hospital, NSW	Royal Melbourne Hospital
Royal Melbourne Hospital	Royal Melbourne Hospital
Alfred Hospital, Melbourne	Alfred Healthcare Group Ethics Committee
Preston & Northcote Community Hospital	Preston & Northcote Community Hospital
Geelong Hospital, Vic	Barwon Health Research & Ethics Committee
Austin & Repatriation Medical Centre (2 sites)	Austin & Repatriation Medical Centre, Austin Campus
Box Hill Hospital, Melbourne	Box Hill Hospital
Dr J Counsell (private practice), Dandenong	Dandenong Hospital Ethics Committee
Gippsland Base Hospital, Vic	Central Wellington Health Service
Dr A Soward (private practice), Mildura	Mildura Private Hospital Ethics Committee
Goulburn Valley Base Hospital, Vic	Goulburn Valley Base Hospital
Dr B Sia (private practice), Melbourne	Royal Melbourne Hospital
Latrobe Regional Hospital (2 sites), Vic	Latrobe Regional Hospital
Wodonga District Hospital, Vic	Royal Melbourne Hospital
Monash Medical Centre, Melbourne	Alfred Hospital Ethics Committee
Wimmera Base Hospital, Vic	Royal Melbourne Hospital
Maroondah Hospital, Melbourne	Maroondah Hospital Ethics Committee
Royal Brisbane Hospital	Royal Brisbane Hospital Ethics Committee
Princess Alexandra Hospital, Brisbane	Princess Alexandra Research Ethics Committee
Toowoomba Hospital, Qld	Toowoomba Health District Research & Ethics Committee
Townsville General Hospital, Qld	Townsville District Health Service Ethics Committee
Rockhampton Hospital, Qld	Rockhampton Hospital Research & Ethics Committee
Redcliffe Hospital, Qld	Redcliffe-Caboolture District Health Service Ethics Committee
Cairns Base Hospital, Qld	Cairns Base Hospital Ethics Committee
Nambour General Hospital,Qld	Royal Brisbane Hospital Ethics Committee
Prince Charles Hospital, Brisbane	Prince Charles Hospital Research & Ethics Committee
Queen Elizabeth Hospital, Brisbane	Princess Alexandra Research Ethics Committee
Gold Coast Hospital, Qld	Gold Coast District Health Service Ethics Committee
Ipswich Hospital, Qld	Ipswich Hospital Research Ethics Advisory Committee

Dr D Colquhoun (2 sites), Brisbane	Greenslopes Private Hospital Ethics Committee;<br>Wesley Hospital Multi-Disciplinary Ethics Committee
Maryborough Hospital,Qld	Fraser Coast Ethics Quality Healthcare Committee
Pindara Specialist Centre, Qld	Gold Coast District Health Service Ethics Committee
Royal Adelaide Hospital	Royal Adelaide Research Ethics Committee
Flinders Medical Centre, Adelaide	Flinders Medical Centre Committee on Clinical Investigation
Port Lincoln Hospital,SA	Flinders Medical Centre Committee on Clinical Investigation
Repatriation General Hospital, Adelaide	Repatriation General Research & Ethics Committee
Queen Elizabeth Hospital, Adelaide	North Western Adelaide Health Service Ethics of Human Research Committee
Sir Charles Gairdner Hospital, Perth	Sir Charles Gairdner Hospital Committee for Human Rights
Royal Perth Hospital	Royal Perth Hospital Ethics Committee
Fremantle Hospital, Perth	Fremantle Hospital Ethics Committee
Royal Hobart Hospital	Royal Hobart Hospital Research & Ethics Committee
Launceston General Hospital, Tas	Launceston Hospital Medical Research Ethics Committee
Auckland Hospital, NZ	North Health Ethics Committee
Christchurch Hospital, NZ	Canterbury Ethics Committee
Dunedin Hospital, NZ	Southern Regional Health Authority Ethics Committee Otago
Gisborne Hospital, NZ	Tairawhiti Ethics Committee
Greenlane Hospital, NZ	North Health Ethics Committee
Memorial Hospital, NZ	Manawutu-Whanganui Ethics Committee
Hutt Hospital, NZ	Wellington Ethics Committee
Middlemore Hospital, NZ	North Health Ethics Committee
Napier Hospital, NZ	Hawkes Bay Ethics Committee
Nelson Hospital	Nelson-Marlborough Ethics Committee
North Shore Hospital, NZ	North Health Ethics Committee
Southland Hospital, NZ	Southern Regional Health Authority Ethics Committee, Southland
Taranaki Hospital, NZ	Taranaki Ethics Committee
Tauranga Hospital, NZ	Bay of Plenty Ethics Committee
Timaru Hospital, NZ	Canterbury Ethics Committee
Waikato Hospital, NZ	Waikato Ethics Committee
Wairau Hospital, NZ	Nelson-Marlborough Ethics Committee
Northland Base Hospital, NZ	North Health Ethics Committee
Wellington Hospital, NZ	Wellington Ethics Committee
Ashburton Hospital, NZ	Canterbury Ethics Committee
