# Supplementary material for: Distributional regression in clinical trials: treatment effects on parameters other than the mean
Source: BMC Med Res Methodol. 2022 Feb 27;22:56. doi: 10.1186/s12874-022-01534-8 (PMC8883706; doi:10.1186/s12874-022-01534-8)
Supplement: Supplementary file 3 — Additional file 3. Supplementary material: Plot of JSU distribution. [file 12874_2022_1534_MOESM3_ESM.pdf]

Supplementary material

Plot of JSU distribution

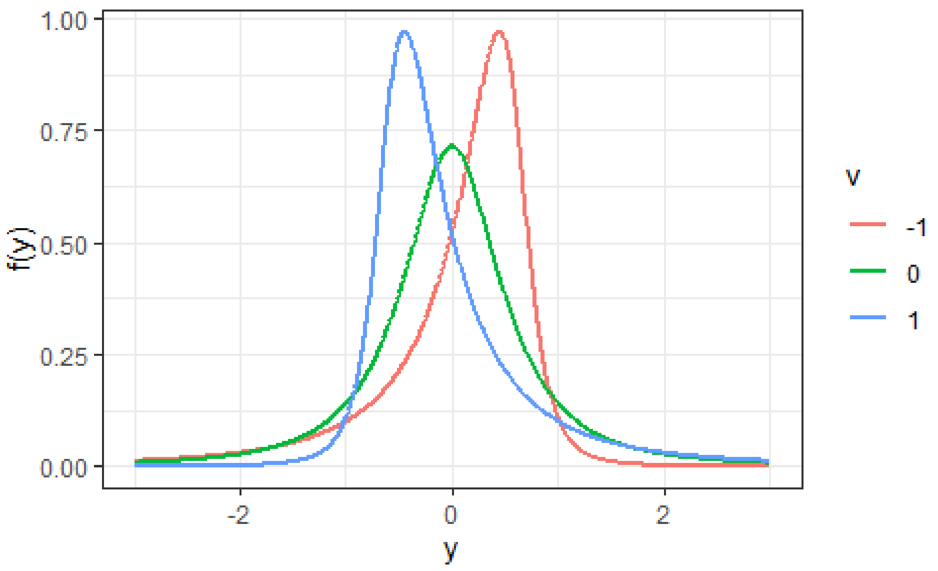

Figure 1: PDF of Johnson's Su (JSU) distribution with μ=0, σ=1, ν=-1,0,1 and τ=1. μ is the mean, σ is the standard deviation, ν controls the skewness and τ is the kurtosis.
